# Cow’s Milk Allergy or Gastroesophageal Reflux Disease—Can We Solve the Dilemma in Infants?

**DOI:** 10.3390/nu13020297

**Published:** 2021-01-21

**Authors:** Silvia Salvatore, Massimo Agosti, Maria Elisabetta Baldassarre, Enza D’Auria, Licia Pensabene, Luana Nosetti, Yvan Vandenplas

**Affiliations:** 1 Department of Medicine and Surgery, Pediatric Unit, “F. Del Ponte” Hospital, University of Insubria, 21100 Varese, Italy; massimo.agosti@uninsubria.it (M.A.); luana.nosetti@uninsubria.it (L.N.); 2 Department of Biomedical Sciences and Human Oncology-Neonatology and NICU Section, “Aldo Moro” University of Bari, 70124 Bari, Italy; mariaelisabetta.baldassarre@uniba.it; 3 Department of Pediatrics, Vittore Buzzi Children’s Hospital, University of Milan, 20154 Milan, Italy; enza.dauria@unimi.it; 4 Department of Medical and Surgical Sciences, Pediatric Unit, University “Magna Graecia” of Catanzaro, 88100 Catanzaro, Italy; licia.pensabene@gmail.com; 5 Kidz Health Castle, Universitair Ziekenhuis Brussel, Vrije Universiteit Brussel, 1090 Brussels, Belgium; yvan.vandenplas@uzbrussel.be

**Keywords:** reflux, GER, GERD, cow’s milk allergy, CMA, eosinophilic esophagitis, infants, hydrolyzed formula, alginate, thickened formula

## Abstract

Cow’s milk allergy (CMA) and gastro-esophageal reflux disease (GERD) may manifest with similar symptoms in infants making the diagnosis challenging. While immediate reaction to cow’s milk protein indicate CMA, regurgitation, vomiting, crying, fussiness, poor appetite, sleep disturbances have been reported in both CMA and GERD and in other conditions such as functional gastrointestinal disorders, eosinophilic esophagitis, anatomic abnormalities, metabolic and neurological diseases. Gastrointestinal manifestations of CMA are often non-IgE mediated and clinical response to cow’s milk free diet is not a proof of immune system involvement. Neither for non-IgE CMA nor for GERD there is a specific symptom or diagnostic test. Oral food challenge, esophageal pH impedance and endoscopy are recommended investigations for a correct clinical classification but they are not always feasible in all infants. As a consequence of the diagnostic difficulty, both over- and under- diagnosis of CMA or GERD may occur. Quite frequently acid inhibitors are empirically started. The aim of this review is to critically update the current knowledge of both conditions during infancy. A practical stepwise approach is proposed to help health care providers to manage infants presenting with persistent regurgitation, vomiting, crying or distress and to solve the clinical dilemma between GERD or CMA.

## 1. Introduction

Gastroesophageal reflux (GER) and cow milk allergy (CMA) occur frequently in the first year of life [1,2,3,4]. The pathogenesis of these two conditions is complex and involves multiple mechanisms of nutrition, motility, immunology and hypersensitivity. A number of papers discussed the overlapping symptoms or simultaneous occurrence of CMA and GERD [1,4,5,6,7,8,9,10,11,12,13,14,15,16,17,18,19,20,21,22,23,24,25,26,27,28,29] Nonetheless, discrimination between both disorders is still challenging due to the similarity of the symptoms and the lack of accurate and handy diagnostic tests [1,27]). Although the response to a CM elimination diet and oral challenge are essential to confirm the diagnosis of CMA [30,31,32,33], a positive challenge test does not proof the involvement of the immune system. Moreover, delayed reactions as occurring in non-IgE mediated allergy, may be insufficiently recognized with an oral challenge test. Upper endoscopy and biopsies and esophageal pH-impedance are the recommended diagnostic investigations for GERD [34]. However, a normal endoscopy and histology does not rule out GERD, as is the case in non-erosive GERD. Normal ranges for pH-impedance are missing and parameters such as symptom association probability have not been validated in children. Performance of pH-impedance is also hampered by cost and investment of time [34,35]. As a consequence, under- or over-diagnosis of CMA and GERD are likely to occur. CM protein elimination diet and treatment with acid inhibitors are often empirically initiated and are, sometimes, excessively protracted.

The aim of this review is to critically update the current knowledge of both conditions during infancy and to provide clinicians a practical stepwise diagnostic and therapeutic approach for infants presenting with persistent regurgitation, vomiting, crying or distress.

## 2. CMA and GERD: A Pathogenic Twist

GER and other persistent gastrointestinal symptoms in allergic patients are predominantly associated with cellular immune mechanisms and delayed reactions. In non-IgE mediated CMA, activated mast-cells, eosinophils and Th2 lymphocytes, release histamine, tryptase, IL-4, IL-5, IL-13, eotaxin and other chemokines that lead to increased permeability, epithelial dysfunction, inflammatory infiltration in the mucosal, submucosal and, in some cases, muscle layers and nociception [25,27,28,36].

A migration of activated mast cells in proximity of enteric nervous system has been demonstrated in allergic children exposed to CM proteins and may determine gastrointestinal dysmotility and related symptoms [37].

GER and regurgitation are commonly related to overfeeding, short length of the (intra-abdominal) esophagus, obtuse His angle, horizontal position of the infant. Inappropriate relaxations of the lower esophageal sphincter (LES), ineffective clearance and the impaired resistance of the esophageal mucosa contribute to GERD [34].

Crying and pain in infants and children are determined by interplaying factors such as esophageal and gastrointestinal distension, dysmotility, visceral hyperalgesia, genetics, early life events, inflammatory and microbiota components, increased permeability, stress, parental and individual coping and perception [4,38,39].

GER and CMA can coexist in the same patient and it has been reported that CMA can induce GER and also be a predisposing factor for gastrointestinal functional disorders [22,27]. Conversely, treatment with acid inhibitors for GERD increase the risk of allergy later in life [40,41].

## 3. Functional Disorder, CMA or GERD: The Clinical Enigma

### 3.1. Definition and Epidemiological Data of Infant Regurgitation and Colic

Infant regurgitation and colic are defined by the Rome IV criteria as functional gastrointestinal disorders (FGIDs) of infancy [42]. Diagnostic criteria for infant regurgitation must include at least due episodes of regurgitation per day for at least three weeks in an otherwise healthy infant 3 weeks to 12 months of age without retching, hematemesis, aspiration, apnea, failure to thrive, feeding or swallowing difficulties or abnormal posturing [42]. Infant colic is defined by recurrent or prolonged periods of crying, fussing or irritability that occur without an obvious cause, that cannot be prevented or resolved by caregivers in an infant younger than 5 months with no failure to thrive, fever or illness [42]. For clinical research purposes, to fulfill the definition of colic these episodes of crying or fussiness should last at least 3 h per days, for a minimum of one day when measured by a prospectively kept 24 h behavior diary or 3 days per week according to a caregiver’s interview [42]. They affect, alone or in combination and depending on selection and inclusion criteria around 20 to 25% of infants all over the world [4,39,43,44]. Neonates born preterm, small for gestational age or exposed to early life antibiotics have been recently reported to be at increased risk of infantile regurgitation and colic [45,46]. One fifth to one third of parents are concerned about their infant’s health condition and consult health care providers because of regurgitation, fussiness and crying [3,4,39,41,47]. Regurgitation and infantile colic occur mostly during the first three to four months of life, with a natural resolution in the vast majority of cases around 4 to 5 months for colic and from 6 months onwards for regurgitation [3,42,48,49,50]. When the onset of regurgitation is in the first two weeks of life or when projectile vomiting is the predominant symptom, secondary GER related to anatomic malformations or conditions such as CMA are more likely [42].

### 3.2. Symptoms and Prevalence of GERD in Infants

When GER is associated with troublesome, persistent severe symptoms or complications (e.g., respiratory problems or esophagitis) it is referred to as GERD [34]. As the definition of troublesome is subjective, the distinction between GER and GERD is challenging in infants and the two terms are often misused interchangeably [34].

The most frequently reported symptom of GER in infants is regurgitation but the latter is neither sensitive nor specific to diagnose GERD, neither if associated with crying or fussiness [14,15,34,38,47,51,52,53]. Thus, acid inhibitors should not be started in these infants unless an investigation-based diagnosis of GERD is established [34]. The exact prevalence of GERD in infants is difficult to define because symptoms are not specific, empirical treatment is often started, many infants are not submitted to pH-impedance and/or endoscopy and prospective data are limited. The only report in which healthy infants (N = 509), screened for risk of sudden infant death syndrome, underwent pH-monitoring dates from 1991 [54]. Using a glass microelectrode to detect acid pH, the 95th percentile of esophageal acid exposure rate, during the first 12 months of life, was about 10% [54]. Hence, 5% of healthy infants, would present a pathological oesophageal acid exposure when the threshold is fixed to 10%. In the last 30 years, for ethical reasons, only symptomatic infants suspected to have GERD were investigated. When 151 infants with persistent crying underwent pH-monitoring, 17.9% infants had pathological acid exposure time (>10%) and no association with total crying duration was noted [15]. Regurgitation occurring more than 5 times daily was the most specific GERD symptom (specificity 70.9%) but had a poor positive predictive value (22%). In the absence of frequent regurgitation or feeding difficulties, pathological GERD according to pH monitoring results was unlikely (negative predictive value 87–90%) [15]. In another study evaluating 100 infants, suspected of having GERD, a pathological pH tracing was found in 21% of cases and esophagitis was identified in 17 out of 44 infants (39%) underwent endoscopy, with poor correlation between clinical symptoms, histology and pH results [51]. In a multicenter retrospective cross-sectional study in the United States using an Endoscopy Database System, emerged that 5.5% of children aged 0 to 1 year had erosive esophagitis [55]. In another cohort of 245 infants with symptoms of reflux submitted to endoscopy and esophageal biopsy, 62 cases (25%) had histological esophagitis [56]. In 8 out of 40 infants (20%) referred for persisting symptoms attributed to GERD (regurgitation and/or vomiting and inconsolable crying, fussiness, irritability, sleeping difficulties or respiratory problems for at least 2 weeks, in the absence of any other identifiable cause) a pathological acid exposure (defined as ≥7%, as measured by an antimony electrode) was found by pH-impedance [57]. More recently, our group analyzed impedance-pH tracings of 62 children (ages 15 days to 23 months, median age 3.5 months) with persistent unexplained fussiness or distress and 19% showed an acid reflux exposure time >7% [58]. 

### 3.3. Symptoms and Prevalence of CMA in Infants

The prevalence of hospital based diagnosed CMA in the first year of life ranges from 0.5% to 3% of infants, with the lowest rate when breast feeding and food challenge are considered [25,28,36,59]. Nonetheless, in a Finnish study, of the 824 exclusively breast-fed infants, 2.1% had CMA, verified by a CM elimination-challenge test [60].

In the EuroPrevall birth cohort study, 12,049 children with symptoms possibly related to CMA were enrolled and 77.5% were followed up to 2 years of age. Clinical evaluation included CM-specific IgE antibodies (IgE), skin prick test and double-blind, placebo-controlled food challenge. CMA was suspected in 358 (3%) children and confirmed by the food challenge in 55 cases (0.54%, 95% CI 0.41–0.70). Of all children with CMA, 23.6% had negative specific serum IgE and all of them tolerated CM one year after diagnosis compared to 57% of those children with IgE-associated CMA [59].

According to these epidemiological data, the expected casual coexistence of CMA and GERD would occur, by far, in less than 1% of the breastfed or formula fed infants. In breastfed infants, reflux and infantile colic as single manifestations are only seldom caused by CMA [61].

GERD may be the cause of regurgitation, vomiting, feeding disorders, day and night crying [34]. Similar symptoms may also be present in CMA and make it difficult to understand which condition is responsible for the clinical picture, especially in the absence of other signs of allergy, such as atopic dermatitis or otherwise unexplained rectal bleeding in the first months of life [1,4,30,31,61,62].

Prolonged crying during or after a meal or in the evening and night are often erroneously attributed to both CMA and GERD which seem to be responsible for only 5–10% of cases of infantile colic [25,27,38].

Repeated episodes of incoercible vomiting, with possible severe dehydration, lethargy and diarrhea occurring within a few hours from CM intake, can be classified as food protein induced enterocolitis syndrome (FPIES) [63,64]. Diarrhea, poor feeding, vomiting, failure to thrive and malabsorption are reported in food protein enteropathy. Food protein induced allergic proctocolitis typically shows he presence of blood and mucous in the stools and mild diarrhea in otherwise well-appearing, often breastfed infants [28,31,32,33,64].

### 3.4. Literature Data on the Association of CMA and GERD

A number of studies examined the presence of CMA in infants with symptoms attributed to GERD (Table 1).

The association of CMA-GERD was reported in 16–56% of cases with persistent gastrointestinal symptoms and suspicion of GERD, irrespective of breast or formula feeding [1,17,23,27,28,29,45,62]. The percentage of infants with persistent GER symptoms with clinical improvement on diet and worsening on challenge is extremely variable depending on the population recruited, design of the study and follow up data [27]. In one study, out of 19 infants with persistent distress and GER symptoms with no response to eHF and acid suppressive agents, 9 infants had esophagitis, 9 had inflammatory changes in the stomach and/or duodenum and all 19 improved on amino acid-based formula [14].

## 4. The Stepwise Approach to Infants with Regurgitation, Vomiting and Crying

**Figure 1:**
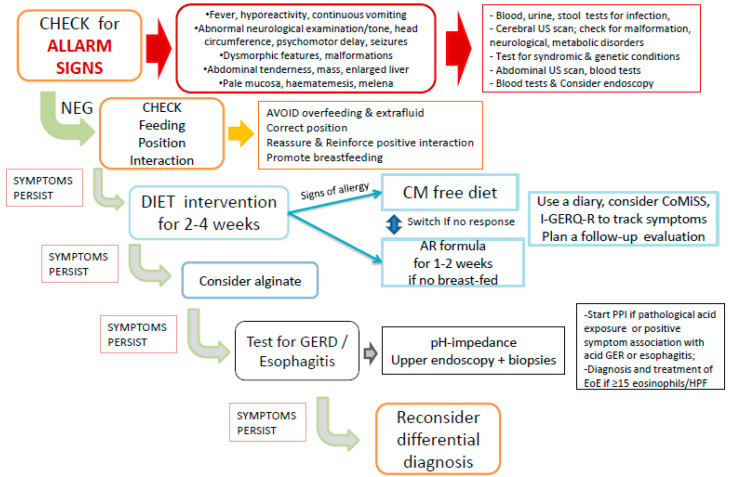
Simplified stepwise approach and ACTION PLAN for infants with persistent (≥1 week) regurgitation, vomiting and crying. See text for complete explanation and further details. Legend: US = ultrasound; CM = cow’s milk protein; AR = thickened; PPI = proton pump inhibitors; GER = gastroesophageal reflux; EoE = eosinophilic esophagitis; HPF = high power field.

In each infant, alarm signals indicative of other conditions such as infectious, neurological, anatomic, surgical, genetic or metabolic pathologies should be excluded throughout an accurate medical history and full physical examination (Figure 1).

Onset of symptoms in the first week or beyond six months of life is not typical of GER. The presence of seizure, psychomotor delay, lethargy or hyporeactivity, abnormal head circumference, abnormal posturing, prolonged inconsolable crying/irritability, muscle hypo/hypertonia or impaired reflexes should alert for neurological or neuromotor or metabolic diseases. Fever, recurrent infections, prolonged apneas, recurrent brief resolved unexplained events (BRUE) or apparent life-threatening events (ALTE), jaundice, pallor, dehydration, bulging or depressed fontanelle, cyanosis, gastrointestinal bleeding, bilious vomiting, abdominal mass or tenderness, hepato/splenomegaly, multiple bruising or hematomas, weight loss or severe failure to thrive should be promptly investigated [34,65]. Abnormal growth, malformations and dysmorphic features should be considered for syndromes and genetic disorders.

Differential diagnosis and specific investigations for these different diseases will not be discussed in this review.

### 4.1. Management of CMA and GER in Infants

In the absence of warning signs, the first step in the management of infants presenting with infantile colic and regurgitation fulfilling the Rome IV criteria is to avoid overfeeding by checking infant’s growth and feeding modalities regarding frequency and duration of feeding and preparation and volume in formula-fed infants. Parental education and information on their infant’s symptoms mechanisms and evolution are of outmost importance [4,34,39]. Reassurance and positive interaction between parents and babies need empathy and patience and should be reinforced [39,65,66,67].

### 4.2. Nutrition, Dietary Modification and Diagnosis of CMA in Infants

Breastfeeding should always be promoted and continued in all infants, even in CMA, functional gastrointestinal disorders and GERD, as human milk represents the best nutritional option. In formula-fed infants, feeding volume and frequency should be progressively adapted according to age and weight and formula changing should be considered in cases with persistent (distressing) symptoms and/or poor weight gain. Commercial thickened formulas provide controlled concentration of various (locust bean gum/carob flour, tapioca, potato, rice, corn starch) thickening agents and nutritional requirements and is likely to decrease the daily episodes of regurgitation by half [66] within the first week.

The National Institute for Health and Care Excellence (NICE) GER- guidelines suggest a greater likelihood of CMA in the presence of regurgitation associated with chronic diarrhea or blood in the stool, other atopic manifestations (eczema) or a positive family history of allergy. In the ESPGHAN guidelines [30,34] the involvement of symptoms in different organ systems in association with the regurgitation increases the likelihood of CMA. Both regurgitation and atopic dermatitis are common disorders in the first months of life and their relation (overlapping age, coincidence or comorbidity) still needs to be further clarified, especially in infants with severe eczema. 

Nonetheless, skin prick tests and specific IgE dosage are positive in only a minority of patients with gastrointestinal symptoms [1,28]. Atopy patch tests and the dosage of specific IgG antibodies are not well standardized and thus not recommended for diagnosing CMA [27,30,31,33]. As a consequence, elimination of CM proteins during 2 to 4 weeks is the recommended approach [30,34].

In breastfed infants, maternal CM free diet can be considered if symptoms are severe enough. In non-breastfed infants with CMA, formulas with CM based extensively hydrolyzed proteins is indicated as first choice, rice hydrolysates are second options and amino acid based formulas (AAF) should be reserved for more severe clinical reactions [28,30,31,32,33,68]. Soy infant formula could be considered in some cases, particularly in infants older than six months and in the absence of severe IgE mediated reactions (e.g., anaphylaxis) and gastrointestinal symptoms [31]. Other milk substitutes (from other mammalian species or plant-based beverages) are not recommended because of possible cross-reactivity, limited studies and scarce evidence of efficacy and nutritional adequacy [69]. Noteworthy, hydrolyzed formulas may vary considerable in terms of source of proteins, method and degree of hydrolysis, macro and micronutrients, additional components (i.e., pre- and probiotics) and proof of clinical benefit [70]. Thus, the results of one particular formula cannot be transferred to a “new” or “similar” one.

In one study, the effect of a thickened and non-thickened casein extensive hydrolyzed formula was analyzed in 72 formula-fed infants (younger than 6 months) with suspected CMA (including persistent unexplained distress or colic, respiratory and/or dermatological symptoms, diarrhea or constipation or blood in the stools and troublesome regurgitation/vomiting of more than five episodes a day) with no previous anaphylactic reactions [24]. The challenge was performed in 52/72 (72%) of the enrolled population and was positive in 65.4%. All cases tolerated both study-formulas and regurgitation was reduced in all infants (6.4 ± 3.2–2.8 ± 2.9, *p* < 0.001). The thickened hydrolysate showed a higher reduction of episodes of regurgitation (−4.2 ± 3.2 regurgitations/day) in infants with both a positive and a negative (−3.9 ± 4.0 regurgitations/day) CM challenge after one month of treatment compared to a minimal effect of the non-thickened hydrolysate (−1.9 ± 3.4 episode of regurgitation) in the group with a negative challenge [24]. The global reduction of a symptom-based score (assessing crying time, number and volume of episodes of regurgitation, consistency of stools, presence and severity of respiratory and dermatological symptoms unrelated to infections), was −7.4 points, with the highest efficacy for the non-thickened hydrolysate in the group with a positive challenge compared to the negative challenge (−9.2 vs. −5.7 points) and versus the thickened formulas between the two groups (−8.1 and −7.1 points) [24]. To better target and assess the effect of a CM free diet, based also on the previous study, a Cow’s Milk Related symptom score (CoMiSS) has been proposed as an “awareness tool” for CMA [71]. This is based on scoring daily duration of crying, number and volume of regurgitation episodes, stool pattern, presence and severity of cutaneous and respiratory manifestations, unrelated to infections. The score ranges from 0 to 33 points [71]. A pooled analysis showed that infants with a CoMiSS > 12 had a 75 % chance to have a positive challenge test [72] and a 89% probability to respond to CM free diet according to another report [73]. In a presumed healthy population of infants, the P95 of the CoMiSS was >9 [73,74]. Despite CoMiSS is an easy tool to help identifying infants who can benefit from CM free diet, it does not replace the need for a diagnostic challenge and still requires further validation studies.

The importance of a clinical re-evaluation after a 2–4 weeks is emphasized both to evaluate the clinical benefit and programming the oral challenge in infants who improved or consider other diagnostic steps for the non-responders (Figure 1). The oral challenge test is required for diagnostic confirmation of CMA, proving a reaction to CM proteins after a clinical response to the exclusion diet [31,32,33,36]. Given the common acquisition of tolerance in the first year of life, particularly in infants with non-IgE allergy [59], diet re-evaluation and reintroduction of CM proteins should be considered and scheduled in order not to prolong unnecessary dietary restrictions. Supervised CM protein challenges are required; hospital setting and time frame, (after 2, 6 or 12 months of diet) should depend on the clinical scenario [30], including symptoms at onset and results of allergic tests [28,31,32,33,36].

The role of food allergy and the benefit of CM free diet in persistent unexplained crying classified as infantile colic are still controversial [3,25,75,76,77]. In an early small trial enrolling 21 colicky infants, CM free diet was superior to parental education and counseling [78]. In another study, enrolling 267 colicky babies, a partially hydrolyzed whey-based formula, containing fructo- and galacto-oligosaccharides and reduced lactose, showed a significant decrease in crying episodes compared to a standard formula after two weeks [79]. In 2010 a systematic review did not report evidence of diet efficacy in colicky infants and highlighted that in most studies data on the reintroduction of normal protein were lacking [75]. However, in 2012, another systematic review analyzed the eleven randomized controlled trials considered to be of good quality and concluded that both breast-fed and formula-fed colicky infants benefited from CM elimination diet [76]. According to the 2018 Cochrane review on dietary modification for infantile colic, including 15 randomized controlled trials and 1121 infants (aged 2 to 16 weeks), a greater reduction in crying time in the intervention group compared to normal CM protein intake was noted in 25% of infants with moderate or severe symptoms in many but not all studies [77]. However, the available studies had small sample sizes and most had a significant risk of bias [77]. 

Furthermore, symptoms such as vomiting, regurgitation and crying can decrease and disappear because of the natural evolution or a placebo effect. Nonetheless, symptoms may reappear when a formula with whole proteins (and normal lactose content) is reintroduced for mechanisms other than the immunological ones of allergy, such as the facilitating effect of gastric emptying of the (partial and extensively) hydrolyzed proteins or less fermentation in the case of a formula with reduced lactose [1,4,27]. Bradigastria and tachigastria have been more frequently detected in patients with CMA than in patients with GER or healthy children [21]. In allergic patients, dysrhythmia, mainly determined by an interaction between eosinophils, mast cells and nerve fibers [37], can impair gastric emptying causing vomiting, increasing reflux and possible pain.

### 4.3. Diagnosis and Treatment of GER and GERD

In both breast-fed and formula-fed infants with persistent regurgitation and distress, aluminum free alginate-based formulations have been reported to significantly reduce the number of episodes of GER and regurgitation and associated symptoms [57,67], with no adverse effects reported in short term trials.

No symptom or cluster of symptoms or questionnaire showed a high sensitivity and specificity for GERD in infants and young children [34,51]. The revised infant GER questionnaire (I-GERQ-R) has a controversial diagnostic value for GERD [29,51,80] but it provides a validated tool to monitor the evolution of symptoms during an intervention trial [80].

The infants who continue to present inconsolable crying and distress, with insufficient improvement after parental reassurance, behavioral and dietetic approaches should be submitted to investigations to identify GERD [34,65].

In some children with GER due to CMA, a particular pH-metric esophageal pattern with a gradual drop in pH after the meal was noted [8]. However, this finding is not present in all infants who respond to the diet and has not been confirmed by other authors [10]. The pH-impedance analysis showed that patients with CMA have predominantly a non-acid GER component [22] that can be even more painful than acid GER [58] but do not benefit from therapy with acid inhibitors.

Several clinical trials, two systematic reviews [47,81], one meta-analysis [82] and pediatric guidelines on GERD [34,83] have shown that treatment with acid inhibitors is not significantly effective in infants with regurgitation or vomiting and/or protracted crying without instrumental evidence of GERD. However, proton pump inhibitors are often empirically prescribed [84] while should be reserved to infants with pathological acid exposure time or significant temporal association between symptoms and acid GER during pH-impedance [34,35] or with evidence of esophagitis [34].

Upper endoscopy is indicated for cases with persistent crying, vomiting, anemia, feeding problems and failure to thrive to identify and characterize esophagitis or enteropathy. Quantification of eosinophils in esophageal biopsies help to differentiate GERD from eosinophilic esophagitis. The presence of villous atrophy and inflammatory infiltrate in the lamina propria on duodenal biopsies is characteristic of patients with CMA [11]. Intestinal permeability tests are also abnormal in these patients [11] but they are not performed in many hospitals, are non-specific and are of limited sensitivity for cases without enteropathy. Contrast X-ray is useful to detect anatomical abnormalities but has no role in diagnosis of GERD [34]. Video fluoroscopy and laryngeal examination by ENT pediatric specialist may identify abnormal swallowing and malformation determining respiratory manifestations. Nevertheless, the presence of laryngeal edema and hyperemia has a limited correlation with pH-impedance results in infants and children [85].

In a recent study 50 infants with persisting crying, vomiting and/or food refusal attributed to CMA and/or GERD were extensively investigated including atopy patch test for CM, milk specific serum IgE antibodies, 48 h cry-fuss diary, I-GERQ-R questionnaire, blinded milk elimination-challenge sequence, 24h pH-impedance monitoring before and after CM elimination, ^13^C-octanoate breath testing for gastric emptying, dual-sugar intestinal permeability, fecal calprotectin and serum vitamin D level, Fourteen infants (28%) were finally diagnosed as CMA. No test or parameter at baseline differentiate infants with and without CMA. Only one infant had positive atopy patch test, none had positive serum IgE and, surprisingly, permeability test was higher in non-CMA infants. In the group with CMA, elimination diet significantly improved GERD symptoms, esophageal clearance and baseline, indirect parameters of esophageal function and mucosal integrity [29].

To quantify the evolution of symptoms and the benefit to the individual patient of any diet or therapeutic intervention, a follow-up visit after 2 weeks should be planned and the evaluation of a daily diary reporting pattern of stools, duration of inconsolable crying, episodes of regurgitation, feeding and sleeping disturbs, CoMiSS and I-GERQ-R scores would be useful to track symptoms.

A simplified stepwise approach and action plan for infants with persistent regurgitation, vomiting and crying is shown in Figure 1.

A correct diagnostic classification is essential to avoid the possible mislabel of “disease” in a “functional” condition or the use of protracted or unnecessary diets [31] or drugs with possible adverse effects [84].

## 5. The Third Wheel: Eosinophilic Esophagitis

The first report of eosinophilic esophagitis (EoE) dates back 1995 [86]. Ten children (median age 5 years, range 8 months–12.5 years), with intractable symptoms attributed to GERD but not responsive to reflux treatment (included Nissen fundoplication in 6 of them), showed improvement (in two patients) or complete resolution (in 8 children) of clinical picture when fed with an amino acid based formula (for at least 6 weeks) and relapsed on challenge. The striking feature was the detection of a high eosinophilic infiltrate (median, 41; range, 15–100) in the esophagus in all cases, with mucosal healing on elemental diet (median, 0.5; range, 0–22) [86]. Since then, EoE has been increasingly recognized at all ages throughout the world. While in children and adolescents dysphagia, bolus impaction, vomiting, epigastric pain and selective feeding can be indicators of EoE, in infants symptoms include regurgitation, vomiting, feeding difficulties, crying, fussiness and poor growth [86,87].

The overlap with CMA not only results from the clinical picture but also from the presence of positive family history of allergy, atopic manifestations and positive allergy tests in about 50% of EoE cases, with a response to a CM and/or other food elimination diet in 70–90% of patients [88].

**Figure 2:**
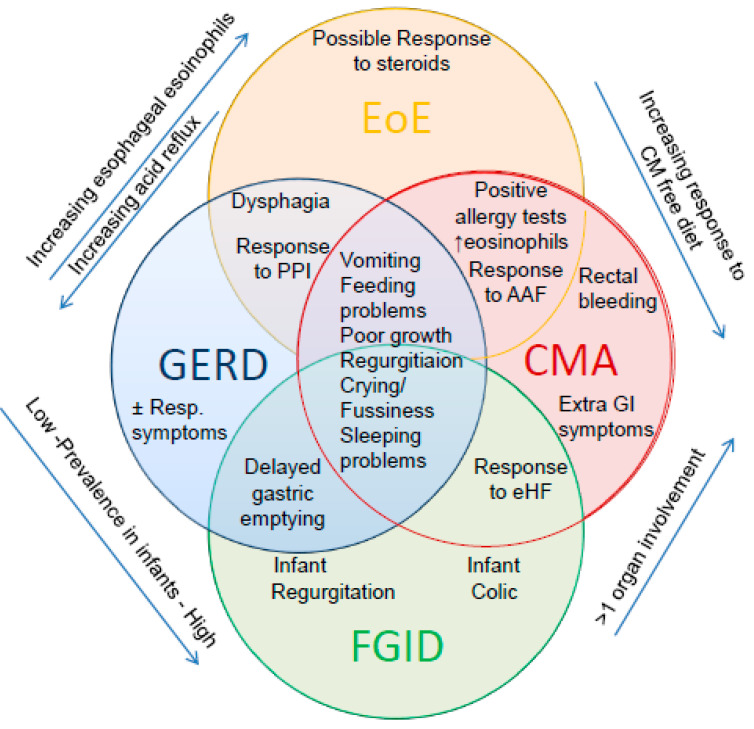
The challenging clinical overlap among Functional Gastrointestinal Disorder (FGID), GERD, CMA and eosinophilic esophagitis (EoE) in infants (modified from Nielsen 2006 [18].

The similarity with GERD is mainly based on the possible reduction of symptoms, acid exposure and esophageal inflammation with PPI [82] (Figure 2). Furthermore, patients with EoE may present a pathological pH-impedance, esophageal dysfunction and stricture [82].

Moreover, CMA, GERD and EoE can all occur with acute, chronic and relapsing manifestations which are difficult to differentiate between the three conditions, particularly in infants and young children [18,26,87,88].

The endoscopic finding of EoE is very variable and can range from normal appearance (particularly in infants) [86] to one or more of the following suggestive but not specific features: food bolus impaction, edema, linear furrows, friability, erosions, ulcerations, concentric rings (up to appearance of trachealization of the lumen), whitish exudates and stricture. The detection of a marked eosinophilic infiltration (>15 by high magnification field, HPF) in at least one esophageal biopsy is the diagnostic hallmark of EoE [87].

The exact prevalence of EoE in both breast- and formula-fed infants [61] is difficult to determine because few infants have endoscopy and esophageal biopsies before been attempted CM free diet or PPI treatment. Moreover, pediatric EoE case series did not provide a subgroup analysis of infants [89] and one large report on infant esophagitis did not detail eosinophilic infiltration [56]. Noteworthy, several early-life factors, including maternal fever, preterm labor, cesarean delivery, esophageal atresia, antibiotic or acid suppressant use in the first months of life, dysbiosis, other atopic conditions and celiac disease have been associated with risk of pediatric EoE [90,91].

The natural history and disease progression of EoE, as well as of CMA and GERD, are not yet well defined because pathogenesis is complex and not fully understood [92]. Therapeutic options for EoE include, as single or sequential intervention: proton pump inhibitors, elimination or elementary diet to avoid allergenic exposure and related inflammation [88]; topical steroids for anti-inflammatory effects; endoscopic dilations for severe stenosis. Immunosuppressive agents and immunomodulators have also been proposed, especially in non-responders adolescents and adults but need further validation [92]. To date, there is no specific, universally accepted and effective treatment for EoE in all patients; consequently, in clinical practice, the therapeutic approach is often individually adapted, especially as regards the choice between dietary or steroid treatment [82,87,92]. On the contrary, in infants the first and, in almost all cases, the only treatment needed for EoE is CM free diet with recommended amino acid formula [31,86,87]. In a recent review of ten studies, enrolling 462 EoE patients (mean age 6.7 years, range, 4 months–20 years), elemental diet resulted in clinical and histological remission (defined as ≤10 eosinophils/hpf) in 75–100% of children [89]. Despite diagnostic difficulties an early recognition of EoE is important to resolve or reduce clinical manifestations and possible long term esophageal complications.

## 6. Conclusions

Persistent regurgitation, vomiting, distress and crying are common symptoms in the first year of life, often coexist in the same patient and can be related to CMA, functional gastrointestinal disorders, GERD, eosinophilic esophagitis and also other different diseases. The real prevalence and the mechanisms underlying the association between CMA and GERD are not yet fully clarified. The lack of an accurate test for non-IgE mediated CMA and for GERD determines the difficulty of a correct diagnostic classification and carries the risk of both delayed recognition and overtreatment. After exclusion of alarm signs for other organic pathologies, a stepwise approach, starting from behavioral and nutritional intervention moving to selected investigations in infants with persistent symptoms could better select infants to start diet and drugs. Because the response to elimination diet, alginate or acid inhibitors may be due to the natural evolution of underlying condition or other than immune or reflux-related mechanisms, periodic reassessment of the patient is essential to avoid misdiagnosis and excessive use of the proposed intervention.

## Figures and Tables

**Table 1:** Summary of the studies evaluating the association of cow’s milk allergy (CMA) and gastro-esophageal reflux disease (GERD) (modified from Ferreira 2014 [23]).

Author, Year	Population	Investigation	Main Results
Forget, 1985 [5]	15 children with recurrent vomiting	Contrast X-ray, small bowel biopsy	All children showed GER on X-ray. 3/15 (20%) had enteropathy with IgE plasmatocytes, reported no improvement with GER treatment but disappearance on symptoms on CM free diet
McLain, 1994 [6]	10 infants with GERD who failed to respond to reflux treatment	pH-monitoring	Symptoms improved in 2/10 (20%) infants on CM free diet. No infant showed significant improvement in pH monitoring indices
Staiano, 1995 [11]	25 infants with recurrent vomiting	Endoscopy and small bowel biopsies, permeability test	Primary GERD in 16/25 (64%), GERD + CMA in 4/25 (16%), CMA alone in 4/25 (16%). Enteropathy in 19% GERD, 67% CMA. Abnormal permeability test in 6% GERD, 100% CMA
Iacono, 1996 [9]	204 infants (median age, 6.3 months) with GERD	pH-monitoring, upper endoscopy, allergy tests, CM challenge	93 (45%) had positive allergy tests, 85 (42%) improved with hydrolyzed formula and reappeared on challenge. GER + CMA significantly associated with the presence of diarrhea or atopic dermatitis
Cavataio, 1996 [8]	96 infants with suspected GERD, CMA and controls	Serum specific IgE and IgG, blood eosinophils, pH-monitoring, endoscopy, CM challenge	14 out of 47 (30%) infants with GERD had CMA These infants had similar symptoms to those with primary GERD but significantly higher concentrations of total IgE, circulating eosinophils and IgG anti-beta lactoglobulin. A specific phasic pH pattern, with progressive decrease in pH tracing, occurred in 24/25 infants with CMA, 12/14 GERD + CMA and 0 controls. CM free diet improved only in the ones with CMA
Milocco, 1997 [10]	112 infants with GERD	pH-monitoring, CM challenge	18 infants (16%) had CMA, 10/18 had failure to thrive. A phasic pH-pattern was present in 1/18 with CMA and in 3 with only GERD
Hill, 2000 [14]	19 infants with persistent distress and GER symptoms with no response to eHF and GERD treatment	Endoscopy, pH-monitoring, CM challenge	Nine infants had histologic evidence of esophagitis and 9 had inflammatory changes in the stomach and/or duodenum. Symptoms remitted in all infants within 2 weeks of starting AAF. On double blind challenge, after a median period of 3 months of AAF, 12 infants were still intolerant to CM
Ravelli, 2001 [21]	26 vomiting infants (7 CMA, 9, GER, 10 controls)	Electrogastrography electrical impedance tomography, CM challenge	Children with CMA showed more gastric dysrythmia (67% vs. 29.4% GER and 30.4% controls) and delayed gastric emptying (89 ± 26 min) compared to infants with GERD (54 ± 13 min) and controls (62 ± 13 min). 7/7 CMA patients had regurgitation and/or vomiting, colic and positive family history of allergy
Garzi, 2002 [12]	10 infants with GER symptoms, 10 controls	Ultrasonography to measure gastric emptying time-with CM formula and protein hydrolysate	All infants with a clinical diagnosis for GER showed delayed gastric emptying vs. normal subjects (205 vs. 124 min, *p* = 0.000). With eHF there was a significant improvement in gastric emptying time and symptoms especially in infants with positive skin-test and RAST
Nielsen, 2004 [17]	18 infants and children (median age 8.7 years; range 2 months to 14.8 years) with GERD	Endoscopy, 48-h pH-metry (Day 1-elimination diet, Day 2-challenge test), 2nd CM challenge	10 (56%) infants had CMA + GERD (higher acid exposure time vs. primary GERD), responded to CM free diet and had a positive challenge which was not associated with a significant increase in the esophageal acid exposure in the simultaneous pH monitoring
Nielsen, 2006 [18]	17 infants and children (aged 2–178 months) (mean age of 7.8 years) with GERD	Endoscopy and biopsies, pH-monitoring, allergy tests, CM challenge	10/17 (59%) were classified as CMA-GERD. Two patients showed >15 eosinophils at biopsies (=EoE) No differences in the number of eosinophils, mast cells or T cells were found between children with CMA and those with primary GERD
Semeniuk, 2007 [19] and 2008 [20]	264 children with suspected GERD (mean age 21 ± 17 months) or CMA	Esophageal manometry, pH-monitoring, allergy tests and CM challenge	138 children with GERD: 76 only GERD, 62 (23.5%) GER + CMA/FA, 32 only CMA/FA. No differences between primary GERD and GERD+ CMA in reflux parameters, in the mean values of resting LES pressure and LES length at baseline and during 2 years of follow-up
Farahmand, 2011 [13]	81 children (aged 1mo-2 yrs, median 12.5 mo) with supsected GERD.	Clinical study	54 (66%) responded to PPI, 27 (33%) to CM elimination diet
Borrelli, 2012 [22]	17 children (median age: 14 months) with proven f CMA and suspected GERD	48-h pH-impedance. Day 1-amino acid formula Day 2-challenge with cow’s milk	The total reflux episodes and the number of weakly acidic episodes were higher during CM challenge compared with the amino acid-based formula period. No differences were found for either acid or weakly alkaline reflux
Vandenplas, 2014 [24]	72 Infants with suspected CMA	Clinical study comparing a thickened and non-thickened eHF casein formula: results after one month.	Regurgitation was reduced in all infants (from 6.4 ± 3.2 to 2.8 ± 2.9, *p* < 0.001) but fell more with the thickened hydrolyzed formula (−4.2 ± 3.2 regurgitations/day) vs. non thickened formula, especially in infants with a negative challenge (−3.9 ± 4.0 vs. −1.9 ± 3.4, ns). In the group with positive challenge the two formulas showed a similar decrease (−4.4 ± 2.6 vs. 4.7 ± 5.6). The global reduction of a symptom-based score was −7.4 points and the non-thickened hydrolysate was more effective in the group with a positive challenge (−9.2 vs. −5.7 points)
Yukselen, 2016 [26]	151 children (aged 3–60 mo) with GERD resistant to 8 wks PPI treatment	skin prick test, specific serum IgE, eosinophil count, atopy patch test and CM challenge	58 children (38.4%) had positive CM challenge and 28 (48%) of them had positive skin prck tests or IgE, 16 (28%) had positive patch tests. Bloody stools, atopic dermatitis and recurrent wheezing episodes were significantly more common in these children Vomiting and diarrhea were more common in non-IgE children. Ten children who had positive challenge were finally diagnosed as EoE
Omari, 2020 [29]	50 infants with persistent crying, vomiting and/or food refusal (suspected to be GERD and or CMA related)	48 h cry-fuss chart, I-GERQ-R, allergy tests, blinded milk elimination-challenge sequence, pH-impedance before and after CM elimination, ^13^C-octanoate breath test for gastric emptying, dual-sugar intestinal permeability, fecal calprotectin	14 (28%) were diagnosed as non-IgE-mediated CMA, 17 (34%) had negative challenge, 19 were excluded for equivocal findings or incomplete data. No baseline differences in any of the tests or GERD parameters between infants with and without CMA. In the CMA group, CM elimination significantly reduced reflux symptoms, esophageal acid exposure, acid clearance time and increased impedance baseline

## Abbreviations

AAF	Amino acid-based formula
CM	cow’s milk
CMA	cow’s milk protein allergy
CoMiSS	cow’s milk related symptom score
EoE	eosinophilic esophagitis
FGIDs	functional gastrointestinal disorders
GER	gastroesophageal reflux
GERD	gastroesophageal reflux disease
I-GERQ-R	revised infant GER questionnaire
PPI	proton pump inhibitors
